# A Bioengineering Strategy to Control ADAM10 Activity in Living Cells

**DOI:** 10.3390/ijms24020917

**Published:** 2023-01-04

**Authors:** Francesco Pastore, Martina Battistoni, Raimondo Sollazzo, Pietro Renna, Fabiola Paciello, Domenica Donatella Li Puma, Eugenio Barone, Onur Dagliyan, Cristian Ripoli, Claudio Grassi

**Affiliations:** 1 Department of Neuroscience, Università Cattolica del Sacro Cuore, 00168 Rome, Italy; 2 Fondazione Policlinico Universitario A. Gemelli IRCCS, 00168 Rome, Italy; 3 Department of Biochemical Sciences “A. Rossi-Fanelli”, Sapienza University of Rome, 00185 Rome, Italy; 4 Division of Molecular Neurobiology, Department of Medical Biochemistry and Biophysics, Karolinska Institute, 171 65 Stockholm, Sweden

**Keywords:** prodomain, cytosolic domain, TEV, engineered protein, APP, Alzheimer

## Abstract

A Disintegrin and Metalloprotease 10, also known as ADAM10, is a cell surface protease ubiquitously expressed in mammalian cells where it cuts several membrane proteins implicated in multiple physiological processes. The dysregulation of ADAM10 expression and function has been implicated in pathological conditions, including Alzheimer’s disease (AD). Although it has been suggested that ADAM10 is expressed as a zymogen and the removal of the prodomain results in its activation, other potential mechanisms for the ADAM10 proteolytic function and activation remain unclear. Another suggested mechanism is post-translational modification of the cytoplasmic domain, which regulates ADAM10-dependent protein ectodomain shedding. Therefore, the precise and temporal activation of ADAM10 is highly desirable to reveal the fine details of ADAM10-mediated cleavage mechanisms and protease-dependent therapeutic applications. Here, we present a strategy to control prodomain and cytosolic tail cleavage to regulate ADAM10 shedding activity without the intervention of small endogenous molecule signaling pathways. We generated a series of engineered ADAM10 analogs containing Tobacco Etch Virus protease (TEV) cleavage site (TEVcs), rendering ADAM10 cleavable by TEV. This strategy revealed that, in the absence of other stimuli, the TEV-mediated removal of the prodomain could not activate ADAM10. However, the TEV-mediated cleavage of the cytosolic domain significantly increased ADAM10 activity. Then, we generated ADAM10 with a minimal constitutively catalytic activity that increased significantly in the presence of TEV or after activating a chemically activatable TEV. Our results revealed a bioengineering strategy for controlling the ADAM10 activity in living cells, paving the way to obtain spatiotemporal control of ADAM10. Finally, we proved that our approach of controlling ADAM10 promoted α-secretase activity and the non-amyloidogenic cleavage of amyloid-β precursor protein (APP), thereby increasing the production of the neuroprotective soluble ectodomain (sAPPα). Our bioengineering strategy has the potential to be exploited as a next-generation gene therapy for AD.

## 1. Introduction

The ADAM (A Disintegrin and Metalloprotease) family includes transmembrane proteins with proteolytic activity on juxtamembrane domains of cell surface substrates implicated in multiple biological processes [1,2,3,4,5]. In humans, the ADAM family includes roughly 30 ADAM isoforms [6], 13 proteolytically active and 11 catalytically inactive and potentially involved in other biological functions, such as protein–protein interaction, axon growth, and neurogenesis [7]. In eukaryotes, ADAM10 and ADAM17 are the main proteases involved in the ectodomain shedding of membrane proteins [8]. For ADAM10, several pieces of evidence suggest that it acts as α-secretase in neurons [9,10,11], participating in non-amyloidogenic cleavage of the amyloid-β precursor protein (APP) [12]. On APP, ADAM10 activity causes the generation of a C-terminal membrane-anchored fragment and a neuroprotective soluble ectodomain (sAPPα), precluding amyloid-β (Aβ) formation and their aggregation leading to amyloid plaque load [13,14,15] that is a hallmark of Alzheimer’s disease (AD). The downregulation of ADAM10 activity is considered a critical factor for AD pathogenesis [13,14], whereas ADAM10 upregulation reduced Aβ-associated pathologies in vitro and in vivo [15,16]. ADAM10 is a component of the excitatory postsynaptic density, where it intervenes in the correct formation and the maintenance of synapses by proteolytic processing of the neurexin–neuroligin complex [17,18,19,20]. Given its involvement in several physiological and pathophysiological processes, ADAM10 is the focus of intense research.

ADAM10 is a transmembrane and multidomain protein of 748 amino acids in length composed of N-terminal signal sequence, prodomain, metalloproteinase domain containing the characteristic HExxHxxGxxH zinc-binding consensus motif, disintegrin domain, cysteine-rich domain, and a cytosolic region [21]. Several studies suggest that the activity of ADAM10 is regulated at the transcriptional, epigenetic, translational, and post-translational levels [22,23,24,25,26].

The post-translational regulation of ADAM10 is mediated by multiple factors, including maturation processes in subcellular compartments, protein interactions, dimerizations, and intracellular second messengers such as Ca^2+^, cAMP, and cGMP [27,28,29]. However, the spatiotemporal dynamics of ADAM10 activation have not been fully identified. ADAM10 has been proposed to be expressed as an inactive zymogen for which the activity is spatially and temporally regulated by endogenous proteolytic activities [30]. It has been suggested that ADAM10 translocates from the endoplasmic reticulum (ER) and trans-Golgi network to the cell membrane, gradually acquiring proteolytic activity [30,31,32]. During its translocation to the cell surface, the progressive processing of ADAM10 by endogenous proteases, such as protein convertase 7 (PC7) and furin, promotes ADAM10 activation. Indeed, the ADAM10 prodomain has been demonstrated to exert potent competitive inhibition of the ADAM10 catalytic domain if applied as a recombinant peptide [33].

The prodomain contains a cleavage site for endogenous proteases (aa 211–215) between the prodomain and the metalloproteinase domain, which is characterized by a dibasic motif RXXR (i.e., RKKR), and it is known as a boundary site (BS) [34]. An additional putative cleavage site has been identified upstream of the BS at residues 48–51 (RAKR), known as the upstream site (US) [34].

Once expressed on the cell surface, ADAM10 forms homodimers through the cytoplasmic tail [35]. Interestingly, homodimerization has been shown to block the catalytic site of the closely related ADAM17, reducing its proteolytic activity [36,37,38,39]. Moreover, the cytoplasmic domain of ADAM10 contains an ER retention motif (i.e., RRR residues 722–724) that keeps ADAM10 in the ER, preventing its surface localization until a stimulus arrives [40,41].

The necessity of different domains in regulating ADAM10 activity has been proposed by using truncated ADAM10 analogs lacking domains, protease-resistant ADAM10 mutants, and pharmacological drugs [21,34,40,42]. However, it remains elusive whether ADAM10 activity could be modulated by controlling the removal of domains from the full-length protein in the absence of physiological stimuli mediating the activation of signaling pathways. Here, we developed a genetically encoded controllable cleavage of the prodomain and cytoplasmic regions of ADAM10, an approach that has been rarely explored as a strategy to control ADAM10 activity in living cells. Using this strategy, we identified an ADAM10 analog with minimum activity in the absence of TEV and significantly higher activity in the presence of TEV. Combining the expression of the identified ADAM10 analogs with a chemically inducible switchable single-chain TEV, we controlled ADAM10 cleavage and activity in space and time. Finally, by using this strategy, we controlled non-amyloidogenic APP processing in living cells, an approach that might be used as a therapeutic strategy for AD.

## 2. Results

### 2.1. Design of Strategy to Control ADAM10 Activity

**Figure 1:**
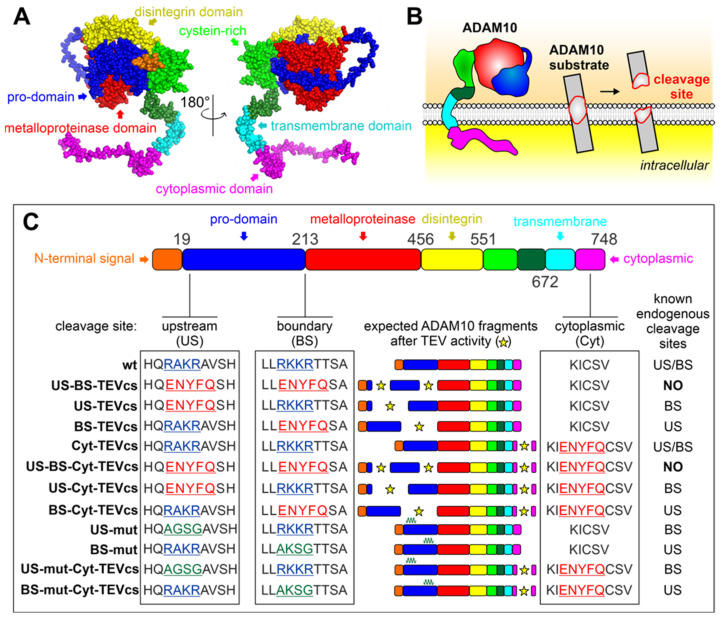
Bioengineering strategy to control ADAM10 activity. (**A**) AlphaFold-predicted the crystal structure of ADAM10 (Uniprot ID: A0A024R5U5). Signaling peptide is shown in orange, prodomain in blue, metalloprotease in red, disintegrin in yellow, cysteine-rich in green, transmembrane in cerulean, and cytoplasmic tail in magenta. (**B**) ADAM10 cleaves cell surface substrates on the juxtamembrane domains. Some shedding fragments are released in the medium. (**C**) Design of strategy to control ADAM10 activity. ADAM10 domains with colors used in (**A**). ENYFQ in red represents TEVcs, AGSG and AKSG in green indicate the mutations in the prodomain. These mutations preclude endogenous protease cleavage. Star indicates the sites of TEV-induced cleavage in the ADAM10 analogs.

The bioengineering strategy proposed here to control ADAM10 activity in living cells is based on the insertion of TEV cleavage site (TEVcs) into specific ADAM10 regions to obtain TEV-mediated cleavage (Figure 1). In particular, we first replaced the two endogenous cleavage sites in the prodomain with TEVcs. This insertion enabled the TEV-mediated ADAM10 protein cleavage to be attained in: (i) US, (ii) BS, and (iii) both regions (Figure 1A–C). We named these constructs: US-TEVcs (on which TEV activity cleaved the prodomain in correspondence with the original US sequence whereas endogenous proteases could process BS), BS-TEVcs (on which TEV activity cleaved the prodomain corresponding to the original BS sequence whereas endogenous proteases could process US), and US-BS-TEVcs (on which TEV processed the prodomain at both the original US and BS) (Figure 1C).

To study the role of the cytosolic region in ADAM10 activity, we introduced TEVcs in the cytoplasmic region (Cyt), generating the Cyt-TEVcs analog (on which TEV activity released a cytoplasmic portion, whereas endogenous proteases could process both US and BS, Figure 1C).

To assess whether the simultaneous cleavage of the cytosolic region and the prodomain affected ADAM10 activity, we generated the following ADAM10 analogs: US-Cyt-TEVcs (TEV cleaved ADAM10 on US and Cyt), BS-Cyt-TEVcs (TEV cleaved ADAM10 on BS and Cyt), US-BS-Cyt-TEVcs (TEV cleaved ADAM10 on US, BS, and Cyt) (Figure 1C). To render ADAM10 insensible to endogenous proteases, we inserted mutations at the endogenous cleavage sites (RAKR mutated with AGSG and RKKR mutated with AKSG in US or BS, respectively) generating US-mut, US-mut-Cyt-TEVcs, BS-mut, and BS-mut-TEVcs (Figure 1C).

All ADAM10 analogs were designed based on the wild-type (wt) sequence of human ADAM10 (Uniprot ID: A0A024R5U5) and contained a Myc-tag at the C-terminus. The overexpression of each ADAM10 analog did not cause cytotoxicity in HEK293T cells. To evaluate TEV-mediated cleavage in SDS-PAGE experiments, we introduced the HA tag in ADAM10 analogs containing Cyt-TEVcs. The activity of ADAM10 analogs was analyzed using the membrane-anchored ADAM10 substrate betacellulin (BTC) fused with the alkaline phosphatase (AP) (BTC-AP). BTC-AP provided a simple and sensitive means to monitor the shedding of ADAM10 in cell-based assays. The activity of ADAM10 on BTC-AP released AP into the medium, which could be analyzed with colorimetric detection [43].

### 2.2. Evaluation of the Role of ADAM10 Prodomain in Shedding Activity

**Figure 2:**
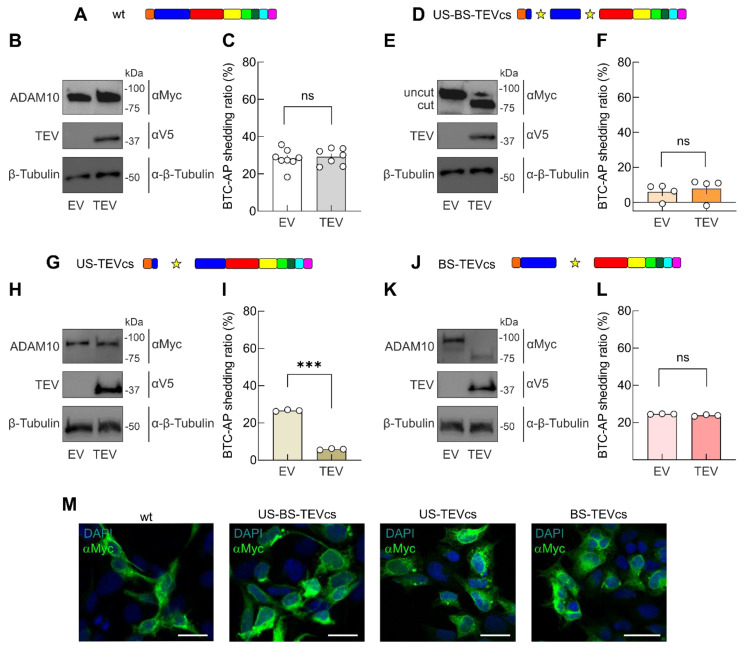
TEV-mediated removal of prodomain does not induce ADAM10 activation. (**A**) ADAM10 protein after TEV activity, wt ADAM10 lacking TEVcs was not a substrate of TEV. HEK293T cells were co-transfected with a ratio of 1:1:1 of plasmids encoding wt ADAM10:BTC-AP:EV (empty vector) or constitutive active TEV. (**B**) Representative WB analysis of cell lysates evaluating the expression and cleavage of wt ADAM10 using an antibody against the C-terminal Myc tag of the expressed proteins. V5 antibody was used to show the presence of TEV. Anti-β-tubulin was used as a loading control. (**C**) Shedding levels in cells expressing EV or TEV were similar. (**D**) US-BS-TEVcs ADAM10 analog after TEV activity. (**E**) Immunoblot analysis revealing TEV-mediated cleavage of US-BS-TEVcs. (**F**) Shedding was unaffected by TEV-mediated cleavage in correspondence of US and BS sites. (**G**) US-TEVcs ADAM10 analog after TEV activity. (**H**) WB of the expression of US-TEVcs analog in the presence of EV or TEV. (**I**) Shedding levels in cells expressing TEV was significantly lower than those in cells expressing EV. (**J**) BS-TEVcs ADAM10 analog after TEV activity. (**K**) Immunoblot analysis of BS-TEVcs analog in the presence of EV or TEV. (**L**) Shedding levels in cells expressing EV or TEV was similar. Star indicates the sites of TEV-induced cleavage in the ADAM10 analogs. Data are expressed as mean ± SEM. ns, not significant; *** *p* < 0.0001; unpaired two-tailed Student’s *t*-test. (**M**) Representative immunofluorescent analysis of ADAM10 analogs in HEK293T cells using antibodies against the C-terminal Myc tag. Scale bar, 25 μm.

To explore the function of the ADAM10 prodomain in controlling protease activity, we evaluated the shedding activity in HEK293T cells transiently transfected with expression plasmids encoding: (i) BTC-AP, (ii) wt or ADAM10 mutants (i.e., US-BS-TEVcs, US-TEVcs, BS-TEVcs), and (iii) an empty vector (EV) or a constitutively active TEV (Figure 2).

As expected, in cells transfected with wt ADAM10 and constitutively active TEV, we did not observe a different SDS-PAGE migration of ADAM10 signal compared to cells transfected with wt ADAM10 and an EV (Figure 2A,B). Similarly, analyzing the levels of BTC-AP, we found no significant differences in AP in the supernatants of cells transfected with wt ADAM10 and TEV compared with wt ADAM10 and EV (shedding activity: 28.21 ± 1.79%, n = 8 vs. 29.19 ± 1.65%, n = 7, in the presence of EV and TEV, respectively, *p* = 0.6973, Figure 2C).

However, in cells expressing US-BS-TEVcs ADAM10 mutant, TEV activity led to an evident migration in SDS-PAGE experiments, reflecting the cleavage of prodomain regions, as expected (Figure 2D,E). When we measured the shedding activity in the supernatants of cells expressing US-BS-TEVcs ADAM10 mutant and EV, we found significantly lower levels of AP detection than cells expressing wt ADAM10 and EV (5.93 ± 2.33%, n = 4, *p* < 0.0001 compared with wt ADAM10). These data indicated that the insertion of TEVcs in correspondence with the original US and BS sequence reduced the shedding activity of ADAM10 (Figure 2F). The inhibition of ADAM10 activity with the US-BS-TEVcs analog could result from the abrogation of endogenous protease activity on the US and BS (Figure 1C). Of note, the shedding activity was unaffected by co-expression of the constitutively active TEV (7.82 ± 3.26%, n = 4, *p* = 0.6531 compared with US-BS-TEVcs + EV, Figure 2F), indicating that the removal of the prodomain without a concomitance of physiological stimuli and/or a differential timing in prodomain processing was ineffective in regulating ADAM10 shedding activity.

To learn more about the potential role of the ADAM10 prodomain in modulating its shedding activity, we transfected cells with the US-TEVcs ADAM10 analog and BTC-AP in the presence of EV or TEV. 

The TEV-mediated cleavage of US-TEVcs ADAM10 analog was evaluated in SDS-PAGE experiments (Figure 2G,H). The shedding ratio for BTC in these cells co-transfected with an EV was similar to those expressing wt ADAM10 and EV (26.64 ± 0.25%, n = 3, *p* = 0.9993 compared with wt ADAM10). Surprisingly, there was a significant reduction in BTC-AP shedding in cells co-transfected with TEV, indicating that ADAM10 cleavage on US negatively modulated ADAM10 protease activity (shedding activity: 5.89 ± 0.22%, n = 3, *p* < 0.0001 compared with US-TEVcs + EV).

Next, we analyzed whether the prodomain processing on BS was required for ADAM10 activation in the absence of other stimuli (Figure 2J). In BS-TEVcs ADAM10-transfected cells, we observed TEV-mediated protein cleavage (Figure 2K) without an increase in BTC-AP shedding compared with cells co-expressing an EV (23.82 ± 0.25%, n = 3, vs. 24.56 ± 0.06%, n = 3, in cells transfected with BS-TEVcs and expressing EV or TEV, respectively, *p* = 0.0449; Figure 2L).

Finally, we stained HEK293T transfected cells with a Myc antibody to analyze the amount of ADAM10 analogs on the cell surface. Figure 2M showed that all constructs were immunostained similarly to wt ADAM10, suggesting that the differences in protease activity are not due to difference in protein expression.

### 2.3. Removal of the Cytoplasmic Domain of ADAM10 Increases Shedding Activity

**Figure 3:**
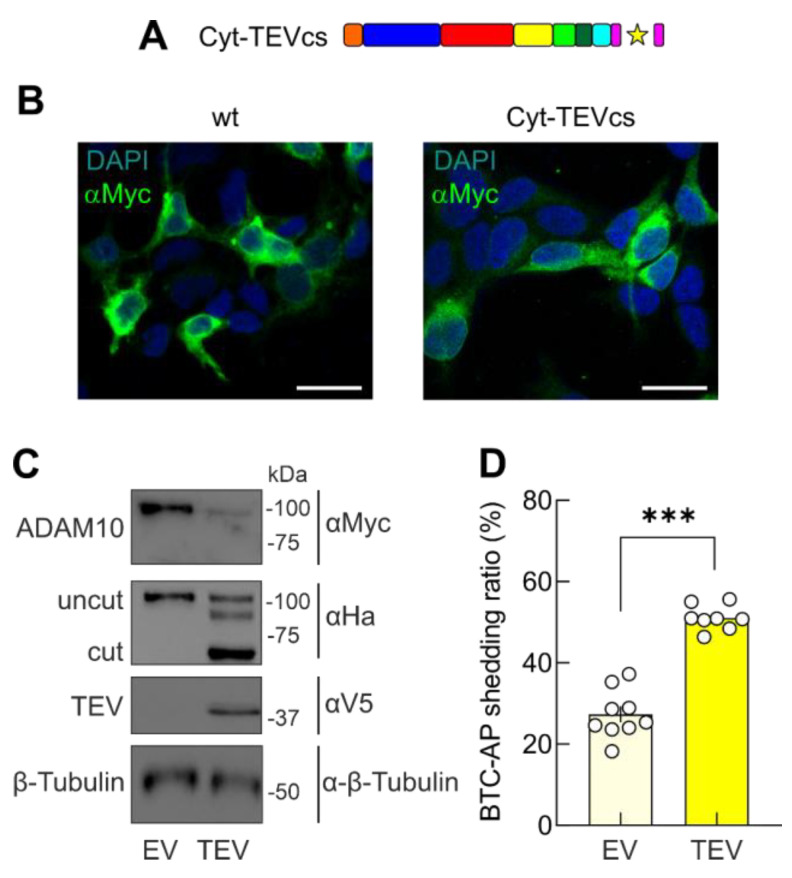
TEV-mediated removal of cytosolic tail increases the constitutive shedding. (**A**) Expected TEV-mediated Cyt-TEVcs ADAM10 resultant cleavage fragments. (**B**) Representative immunofluorescent analysis of Cyt-TEVcs ADAM10 expressing cells using Myc antibody. Scale bar, 25 μm. (**C**) Representative WB analysis of cell lysates evaluating the expression and cleavage of Cyt-TEVcs ADAM10 using antibodies against the C-terminal Myc and C-terminal HA tags of the expressed proteins. Immunoblots with V5 and β-tubulin antibodies were used to evaluate the presence of TEV and the loading amount of lysates, respectively. (**D**) Cells were analyzed for BTC-AP release in the presence EV and TEV. The Cyt-TEVcs ADAM10 analog cleaved by TEV had significantly higher shedding activity. Star indicates the sites of TEV-induced cleavage in the ADAM10 analogs. Data are expressed as mean ± SEM. *** *p* < 0.0001; unpaired two-tailed Student’s *t*-test.

To evaluate the role of the cytoplasmic tail in controlling ADAM10 activity, we co-transfected HEK293T cells with BTC-AP, Cyt-TEVcs ADAM10, and EV or a constitutively active TEV. The cytoplasmic tail has been demonstrated to influence ADAM10 membrane trafficking [41] through an ER retention motif (i.e., 723-RRR) that negatively regulates ADAM10 membrane localization by keeping the protein in the ER [40]. In our Cyt-TEVcs ADAM10 analog, we inserted the TEVcs at the beginning of the cytoplasmic domain (694-GFIKI) upstream of the 723-RRR motif (Figure 1C and Figure 3A). In the upstream sequence of TEVcs, we introduced an HA tag to evaluate the cleavage in SDS-PAGE experiments. In immunofluorescence experiments, we found that Cyt-TEVcs ADAM10 analog immunoreactivity was similar to that of wt ADAM10 in HEK293T cells (Figure 3B). The appearance of a 90 kDa band revealed with an HA antibody demonstrated that TEV-mediated Cyt-TEVcs ADAM10 released the cytoplasmic tail (Figure 3C). When we evaluated the shedding activity of Cyt-TEVcs ADAM10 mutant in cells co-transfected with an EV, we found similar background shedding activity to the wt ADAM10 (27.33 ± 1.99%, n = 9, *p* = 0.9994 compared with wt ADAM10). However, in cells transfected with Cyt-TEVcs and TEV, we observed a significant increase in ADAM10-mediated AP shedding, demonstrating that the TEV-mediated removal of cytosolic domain significantly increased ADAM10 activity (51.07 ± 1.09%, n = 8, *p* < 0.0001 compared with Cyt-TEVcs + EV; Figure 3D).

### 2.4. Developing ADAM10 Analogs with Minimum Activity in the Absence of TEV and Maximum Activity in the Presence of TEV

Because we aimed to obtain minimum activity in the absence of TEV and maximum activity in the presence of TEV, as expected from an ideal engineered protein switch, we intervened on ADAM10 to lower the basal catalytic activity to a minimum.

Having demonstrated that the insertion of TEVcs in the prodomain, in correspondence with the original US and BS sequence, drastically reduced the shedding activity of ADAM10 (Figure 2F), we tested the role of a concomitant cut in pro- and cytoplasmic domains using the US-BS-Cyt-TEVcs mutant (Figure 1C).

**Figure 4:**
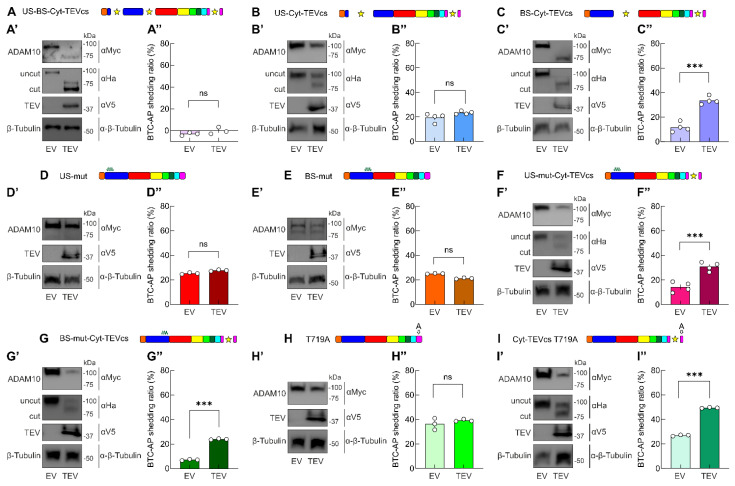
TEV-mediated shedding activity of ADAM10 analogs with mutations in the prodomain and cytosolic region. (**A**) ADAM10 protein after TEV activation. (**A′**) Representative WB analysis of cell lysates evaluating the expression and cleavage of US-BS-Cyt-TEVcs. (**A″**) Shedding levels in cells expressing EV were almost zero and TEV did not increase constitutive shedding. (**B**) US-Cyt-TEVcs ADAM10 analog after TEV activity. (**B′**) Immunoblot analysis revealing TEV-mediated cleavage of US-Cyt-TEVcs. (**B″**) Shedding was unaffected by TEV-mediated cleavage in corresponding US and Cyt sites. (**C**) BS-Cyt-TEVcs ADAM10 analog after TEV activation. (**C′**) WB of the expression of BS-Cyt-TEVcs analog in the presence of EV or TEV. (**C″**) Shedding levels in cells expressing TEV were significantly higher than those in cells expressing EV. (**D**,**E**) ADAM10 proteins after TEV activation; US-mut and BS-mut ADAM10 analogs lacking TEVcs were not TEV substrates. (**D′**,**E′**) Immunoblot analysis in cells expressing US-mut (**D′**) and BS-mut (**E′**) in the presence of EV and TEV. (**D″**,**E″**) Shedding levels in cells expressing EV or TEV for US-mut (**D″**) and BS-mut (**E″**). (**F**,**G**) US-mut-Cyt-TEVcs and BS-mut-Cyt-TEVcs ADAM10 proteins after TEV activation. (**F′**,**G′**) Immunoblot analysis in cells expressing US-mut-Cyt-TEVcs (**F′**) and BS-mut-Cyt-TEVcs (**G′**) in the presence of EV and TEV. (**F″**,**G″**) Shedding levels in cells expressing EV or TEV for US-mut-Cyt-TEVcs (**F″**) and BS-mut-Cyt-TEVcs (**G″**). (**H**,**I**) T719A and T719A Cyt-TEVcs ADAM10 proteins after TEV activation. (**H′**,**I′**) Representative WB analysis of cell lysates evaluating the expression and cleavage of T719A (**H′**) and T719A Cyt-TEVcs (**I′**). T719A analog lacking TEVcs was not a TEV substrate. (**H″**,**I″**) Shedding levels in cells expressing EV or TEV for T719A (**H″**) and T719A Cyt-TEVcs (**I″**). Star indicates the sites of TEV-induced cleavage in the ADAM10 analogs. Data are expressed as mean ± SEM. ns, not significant; *** *p* < 0.0001; unpaired two-tailed Student’s *t*-test.

When we evaluated the shedding activity of US-BS-Cyt-TEVcs analog in cells co-transfected with EV, we found that this mutant was unable to cut BTC-AP (shedding activity: −3.08 ± 0.79%, n = 3, *p* < 0.0001 compared with wt ADAM10, Figure 4A–A″). The co-expression of TEV produced a different SDS-PAGE migration of ADAM10 signal compared to cells transfected with US-BS-Cyt-TEVcs and EV (Figure 4A,A′). However, cleaved US-BS-Cyt-TEVcs analog did not increase its shedding activity, indicating that concomitant TEV-mediated cleavage in pro- and cytoplasmic domains was ineffective in promoting ADAM10 catalytic activity (−0.13 ± 1.81%, n = 3, *p* = 0.2096 compared with US-BS-Cyt-TEVcs + EV; Figure 4A″).

To further study the effects of the removal of the cytoplasmic domains with the concomitant cleavage of one of the prodomain sites, we generated the US-Cyt-TEVcs and BS-Cyt-TEVcs ADAM10 analogs. We found that in US-Cyt-TEVcs transfected cells, TEV promoted ADAM10 analog cleavage without modulating the release of AP (19.46 ± 1.76%, n = 4, vs. 23.4 ± 0.66%, n = 4, in cells transfected with BS-TEVcs and expressing EV or TEV, respectively, *p* = 0.0815, Figure 4B–B**″**). Moreover, the background shedding activity of US-Cyt-TEVcs mutant in cells co-transfected with EV was similar to those of the wt ADAM10 (*p* = 0.0125 compared with wt ADAM10 + EV).

However, we observed a significantly decreased shedding of BTC from cells co-transfected with BS-Cyt-TEVcs ADAM10 and EV compared with wt ADAM10 and EV (11.75 ± 1.94%, n = 4, *p* < 0.0001). Interestingly, when we co-transfected BS-Cyt-TEVcs ADAM10 with TEV, we found a significant increase in the shedding of BTC-AP compared with BS-Cyt-TEVcs ADAM10 co-transfected with EV (33.53 ± 1.63%, n = 4, *p* = 0.0001, Figure 4C–C**″**). These results indicated that the insertion of TEVcs in correspondence with the original BS sequence and the cytoplasmic tail was useful in reducing the background activity of ADAM10 and maintaining TEV-mediated catalytic inducibility.

To further dissect the individual roles of US and BS in ADAM10 catalytic activity, we inserted mutations (i.e., AGSG and AKGS in US and BS, respectively) to block endogenous cleavage at one of these two sites. Mutations in US did not change the shedding activity compared to wt ADAM10 also in cells co-expressing TEV (shedding activity US-mut + EV: 25.25 ± 0.4%, n = 3, *p* = 0.9654 compared with wt ADAM10; US-mut + TEV: 27.67 ± 0.37%, n = 3, *p* = 0.0111 compared with US-mut + EV; Figure 4D–D**″**). A similar picture emerged when we used the BS-mut analog (shedding activity BS-mut + EV: 25.21 ± 0.14%, n = 3, *p* = 0.9627 compared with wt ADAM10; BS-mut + TEV: 21.33 ± 0.3%, n = 3, *p* = 0.0003 compared with BS-mut + EV; Figure 4E–E**″**).

Next, we used US-mut-Cyt-TEVcs (on which TEV activity released a cytoplasmic portion, whereas endogenous proteases could process only BS, Figure 1C) and BS-mut-Cyt-TEVcs (on which TEV activity released a cytoplasmic tail, whereas endogenous proteases could process US but not BS, Figure 1C). With both constructs, we observed a significant decrement of shedding activity compared with wt ADAM10 (*p* < 0.0001 and *p* < 0.0001 for US-mut-Cyt-TEVcs + EV and BS-mut-Cyt-TEVcs + EV, respectively) and a significant increment in shedding levels for BTC in cells co-transfected with TEV (shedding activity US-mut-Cyt-TEVcs + EV: 14.34 ± 2.06%, n = 4 vs. US-mut-Cyt-TEVcs + TEV: 30.9 ± 1.81%, n = 4, *p* = 0.0009; BS-mut-Cyt-TEVcs + EV: 7.15 ± 0.28%, n = 3 vs. BS-mut-Cyt-TEVcs + TEV: 23.99 ± 0.24%, n = 3, *p* = <0.0001; Figure 4F–G**″**).

Another strategy we used to decrease the basal catalytic activity of ADAM10 included the point mutation of a threonine at position 719 (T719) in the cytoplasmic tail. It has been proposed that the catalytic activity of ADAM10 is also regulated through the inhibition of its dimerization by means of phosphorylation on T719. For this reason, we generated a T719 phosphoblocking ADAM10 mutant in which T719 was mutated to alanine (T719A). We reasoned that by blocking T719 phosphorylation, we promoted ADAM10 dimerization, thus reducing ADAM10 constitutive catalytic activity. T719 is located downstream of TEVcs in Cyt-TEV ADAM10 analog; thus, TEV activity could potentially abrogate dimerization by cutting the dimerization sites.

To evaluate the role of T719A in ADAM10 activity, we first transfected HEK293T cells with T719A ADAM10 mutant and BTC-AP. We found similar background shedding activity of T719A ADAM10 mutant to the wt ADAM10 (36.3 ± 2.71%, n = 3, *p* = 0.0573 compared with wt ADAM10). Additionally, the shedding activity of T719A is not affected by TEV (39.22 ± 0.55%, n = 3, *p* = 0.3512 compared with T719A + EV). Similarly, the T719A mutation in Cyt-TEVcs ADAM10 analog did not significantly reduce background activity on BTC-AP substrate (shedding activity T719A Cyt-TEVcs ADAM10 mutant + EV: 26.94 ± 0.3%, n = 3, *p* = 0.9994 compared with wt ADAM10). However, in the presence of TEV, shedding activity was significantly increased (T719A Cyt-TEVcs ADAM10 mutant + TEV: 49.46 ± 0.24%, n = 3, *p* < 0.0001 compared with T719A Cyt-TEVcs ADAM10 mutant + EV; Figure 4H–I**″**).

**Figure 5:**
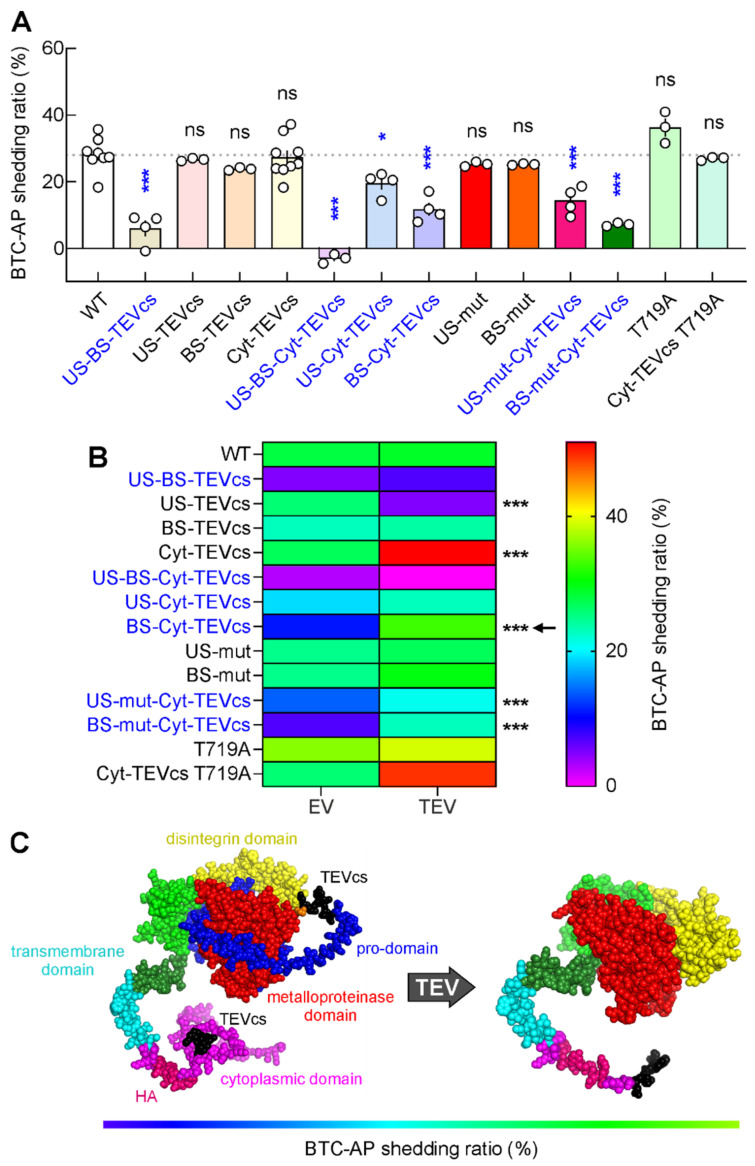
Evaluating ADAM10 analogs with minimum activity in the absence of TEV and maximum activity in the presence of TEV. (**A**) Comparative analysis of constitutive shedding activity of ADAM10 analogs in the presence of EV. Data are expressed as mean ± SEM. ns, not significant compared to WT; * *p* < 0.05 compared to WT; *** *p* < 0.0001 compared to WT; one-way ANOVA with the Dunnett’s post hoc test comparisons. (**B**) Heat map of results of shedding activity of ADAM10 analogs in the presence of EV and TEV. *** *p* < 0.0001; unpaired two-tailed Student’s *t*-test. Arrow indicates the construct generating the minimum shedding activity in the absence of TEV (EV) and a significant increase in BTC-AP shedding in the presence of TEV. (**C**) AlphaFold-predicted crystal structure of BS-Cyt-TEVcs ADAM10 analog before (left) and after (right) TEV activity.

Among all constructs, we identified BS-Cyt-TEVcs as the analog with minimal basal activity in the absence of TEV and maximum activity in the presence of TEV. Indeed, when expressed with (i) EV, BS-Cyt-TEVcs led to a significant reduction in basal shedding activity compared with wt ADAM10, and with (ii) TEV, BS-Cyt-TEVcs shedding activity was significantly increased (Figure 4G–G**″** and Figure 5B).

### 2.5. A Chemogenetic Strategy to Control ADAM10 Activity in Living Cells

**Figure 6:**
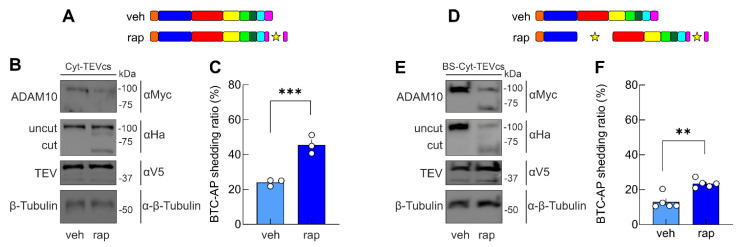
Chemogenetic control of ADAM10 analog cleavage and activation. (**A**) Schematic drawing of Cyt-TEVcs ADAM10 before (veh) and after (rap) uniRapR-TEV activation. (**B**) Assessment of Cyt-TEVcs ADAM10 cleavage by WB analysis of lysates from HEK293T cells transfected with Cyt-TEVcs and uniRapR-TEV and treated with veh or rap. (**C**) uniRapR-TEV activation significantly increased Cyt-TEVcs shedding. (**D**) Schematic drawing of BS-Cyt-TEVcs before (veh) and after (rap) uniRapR-TEV activation. (**E**) Cleavage of BS-Cyt-TEVcs by uniRapR-secTEV assessed by WB. (**F**) In the presence of rap, a significant increase of BS-Cyt-TEVcs shedding was observed, indicating that uniRapR-TEV-mediated cleavage activated ADAM10 analog. Star indicates the sites of TEV-induced cleavage in the ADAM10 analogs. Data are expressed as mean ± SEM. ** *p* < 0.001; *** *p* < 0.0001; unpaired two-tailed Student’s *t*-test.

We recently developed a genetically encoded switchable single-chain TEV, allowing the inducible cleavage of proteins containing the TEVcs in different compartments of cells using rapamycin or its non-immunosuppressive analogs [44]. Here, we used this controllable TEV (uniRapR-TEV) to attempt the chemogenetic modulation of TEVcs ADAM10 analogs activity. Rapamycin did not affect BTC-AP shedding in HEK293T cells transfected with BTC-AP alone compared with cells transfected with BTC-AP and treated with vehicle (*p* = 0.2673). Similarly, the co-expression of uniRapR-TEV and Cyt-TEVcs did not affect ADAM10 cleavage when cells were treated with vehicle (Figure 6A,B). However, rapamycin promoted uniRapR-TEV activation and the removal of Cyt region, increasing the catalytic activity as demonstrated by monitoring BTC-AP shedding (24.01 ± 1.1%, n = 3 vs. 45.61 ± 3.06%, n = 3, in the presence of vehicle and rapamycin, respectively, *p* = 0.0027, Figure 6A–C). Similarly, the rapamycin-mediated activation of uniRapR-TEV induced the proteolytic processing of BS-Cyt-TEVcs, increasing its shedding activity (13.03 ± 1.89%, n = 5 vs. 23.52 ± 1.08%, n = 5, in the presence of vehicle and rapamycin, respectively, *p* = 0.013, Figure 6D–F). These results show that our strategy enables effective control of ADAM10 activity in living cells with rapamycin, which is an inert compound on the timescale of our experimental manipulations.

### 2.6. Controllable ADAM10 Activation Increases α-Secretase Activity and Processing of APP

**Figure 7:**
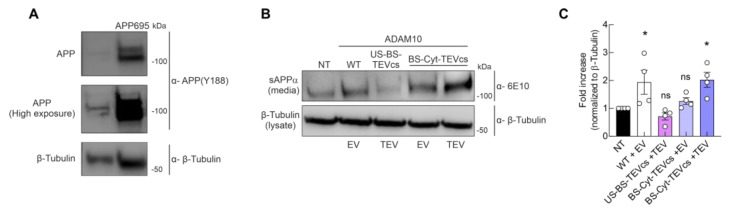
TEV-mediated ADAM10 activation increases non-amyloidogenic processing of APP. (**A**) HEK293 cells stably overexpressing had higher levels of APP in cell lysates compared with wt HEK293T cells. Cell lysates were analyzed by immunoblotting for APP using Y188 antibody. β-Tubulin was used as a loading control. (**B**) APP695 HEK293 cells stably overexpressing APP695 were transiently transfected with wt, US-BS-TEVcs and TEV, or BS-Cyt-TEVcs in the presence of EV or TEV. NT, not transfected. The conditioned medium and cell lysates were analyzed by immunoblotting for sAPPα using antibody 6E10 and β-tubulin was used as a loading control. (**C**) Densitometric quantification of conditions described in (**B**). Data are expressed as mean ± SEM. * *p* < 0.05 compared to NT; ns, not significant compared to WT. Statistical analysis by one-way ANOVA with Dunnett’s post hoc test comparisons.

Finally, we tested whether our bioengineering strategy could be useful to promote the non-amyloidogenic processing of APP. To this aim, we used cells stably overexpressing APP695 with Swedish mutations. We transiently transfected cells overexpressing APP695 with one of the following combinations of cDNA: (i) wt ADAM10 + EV; (ii) US-BS-TEVcs + TEV as negative control; (iii) BS-Cyt-TEVcs + EV; and (iv) BS-Cyt-TEVcs + TEV. The cell growth medium was analyzed to detect ADAM10-dependent release of soluble αAPP (sAPPα) fragment derived from α-secretase-mediated APP processing [45,46]. In Western blot analyses, we detected similar amounts of lysate loading control protein, allowing for a comparative analysis of its α-secretase activity on APP processing (Figure 7A). In cells transiently transfected with wt ADAM10, we observed a significant increase in sAPPα compared with untransfected (NT) cells (1.94 ± 0.43, n = 4, *p* = 0.0471 compared with NT, Figure 7B). However, as expected, in cells transfected with the ADAM10 analog without activity on BTC-AP (i.e., US-BS-TEVcs ADAM10 mutant, Figure 2D–F), we found no significant differences in the release of sAPPα compared with NT cells (0.72 ± 0.13, n = 4, *p* = 0.8202, Figure 7B). These results suggest the reliability of this approach in monitoring ADAM10-mediated APP processing. Thus, we investigated whether the above-identified ADAM10 mutant differentially cut APP in the presence of TEV. The co-expression of BS-Cyt-TEVcs and EV did not show a significant difference in sAPPα release compared to NT (1.26 ± 1.12, n = 4, *p* = 0.8572, Figure 7B). Consistent with our findings for BTC-AP shedding, the co-expression of BS-Cyt-TEVcs and TEV significantly increased the amount of sAPPα in the cell medium (2.03 ± 0.27, n = 4, *p* = 0.0053 Figure 7B), indicating that our bioengineering strategy is useful for controlling the non-amyloidogenic cleavage of APP.

## 3. Discussion

In mammals, ADAM10 is ubiquitously expressed for the proteolytic cleavage of several cell surface proteins implicated in multiple physiological processes [47]. The dysregulation of ADAM10 expression and function has important roles in the development of pathological conditions, including AD, autoimmune disorders, and cancer [14,48,49].

Although ADAM10 appears to be a promising therapeutic target for a wide range of diseases, much remains to be understood about the mechanisms of action of ADAM10 proteolytic function. Experimental evidence suggests that the regulation of ADAM10 occurs in a very complex manner at the transcriptional, post-transcriptional, translational, and post-translational levels [50]. ADAM10 is finely regulated at the post-translational level by the activity of multiple factors. Once expressed and matured by the action of endogenous proteases, ADAM10 is catalytically active but not at full enzymatic function because it acquires a closed conformational state involving the cysteine-rich domain precluding the access of substrates to the active site [21]. Moreover, Ca^2+^, cAMP, and cGMP, affecting the ADAM10 cytoplasmic and transmembrane domains, promote ADAM10 structural activation and its efficient substrate cleavage [51]. Indeed, Ca^2+^ influx elicitable by treatment of cells with ionophores, purinergic receptor agonists, or membrane-perturbating agents induces a scramblase-dependent externalization of the negatively charged phospholipid phosphatidylserine, which positively regulate ADAM10 activity [52].

Here, we report the engineering of the ADAM10 protein to regulate the removal of the prodomain and/or cytosolic region with the aim of achieving spatiotemporal control of ADAM10 activity independently of endogenous signaling pathways.

To this end, we mutated the consensus RX(K/R)R sequences in the prodomain with ENYFQS (i.e., TEVcs). Replacement of cleavage sites of endogenous proteases with TEVcs renders ADAM10 a TEV substrate and, at the same time, insensible to endogenous proteases on mutated sites. We also introduced TEVcs at the beginning of the cytoplasmic domain (694-GFIKI) upstream of the 723-RRR ER retention motif, which is known to negatively regulate ADAM10 membrane localization and activity [40,41]. The co-expression of a constitutive active TEV, exhibiting high specificity for TEVcs and negligible activity toward endogenous mammalian proteomes, ensures a post-translational ADAM10 cleavage in correspondence with desired sequences. The co-expression of BTC-AP, an ADAM10 analog, and TEV allowed us to evaluate the shedding activity of ADAM10 by analyzing the amount of AP released into the medium.

Our results demonstrated that the replacement of the consensus RX(K/R)R sequence with TEVcs in both US and BS significantly reduced the basal catalytic activity of ADAM10 compared with wt ADAM10 (Figure 2D–F and Figure 5). Moreover, our results are in line with other observations indicating that cleavage sites for proprotein convertases are mandatory for producing the catalytically active ADAM10 [32]. The removal of the TEV-mediated prodomain did not affect the ability of the ADAM10 analog to shed BTC-AP (Figure 2D–F and Figure 5), suggesting that the removal of the prodomain required a time-lag cleavage of US and BS or their processing in a different subcellular compartment. Moreover, US and BS replacements with TEVcs could affect the prodomain sequence to such an extent that these mutations influenced the intramolecular chaperone for correct folding, as suggested by Anders and colleagues [32]. However, the replacement of TEVcs in the prodomain corresponding to one of the two sites did not affect the basal shedding activity of ADAM10 analogs compared with wt ADAM10 (Figure 2G–L and Figure 5), potentially indicating that, in these constructs, protein folding was not affected and/or that the action of endogenous proteases at one of the two sites is sufficient to promote basal activation of ADAM10. However, TEV-mediated cleavage on US, but not on BS, significantly blocked the shedding activity of ADAM10, potentially reflecting a competitive inhibition action of the fragment with its catalytic domain [33,34] (Figure 2G–L and Figure 5). Thus, we found that TEV-mediated prodomain removal in US or in both sites did not activate ADAM10.

However, our results demonstrated that inducing the removal of the cytoplasmic tail, upstream of the cytoplasmic ER retention motif, was a strategy to modulate protease activity unless at least one of the two sites (US or BS) in the prodomain was not mutated (Figure 3, Figure 4 and Figure 5). The importance of the cytosolic domain on ADAM10 activity has already been demonstrated by using ADAM10 lacking the cytosolic tail. This mutant showed significantly higher activity compared with the full-length protein, maintaining the sensitivity to endogenous messengers [40].

It has been proposed that ADAM10 is expressed at the cell surface as dimers through its cytoplasmic tail and that dimerization inhibits the catalytic activity [35]. Dimerization is inhibited by threonine phosphorylation occurring in the C-terminal region and, in turn, catalytic activity increases [36,39]. Although the control of dimerization and activity through phosphorylation of T735 has been demonstrated in ADAM17, it remains to be shown whether the phosphorylation of a conserved threonine at position 719 in ADAM10 controls its dimerization and catalytic activity [38]. Our results demonstrated that the T719A and T719A Cyt-TEVcs ADAM10 mutants behaved similarly to wt ADAM10 (Figure 4 and Figure 5), suggesting that basal catalytic activity, in the absence of stimuli, was unaffected by the loss of the phosphorylation on T719. Similarly, in the T719A Cyt-TEVcs mutant, the TEV-mediated release of the cytosolic tail significantly increased the shedding of BTC-AP, confirming once again that the removal of the cytoplasmic tail was responsible for an increase in the catalytic activity of ADAM10 (Figure 4 and Figure 5).

Moreover, using the ADAM10 constructs developed here, we found that the simultaneous perturbation of the ADAM10 sequence at two sites significantly reduced the catalytic activity of ADAM10. Indeed, a significant reduction in the shedding of BTC-AP was observed for ADAM10 analogs containing the replacement of US + BS, US + Cyt, and BS + Cyt (Figure 2, Figure 4, and Figure 5). When all three sites were mutated (US + BS + Cyt), we observed a drastic collapse in the catalytic activity (Figure 4 and Figure 5).

Of all the generated constructs, the one in which we inserted TEVcs corresponding to both BS in the prodomain and upstream of the cytoplasmic ER retention motif rendered an ADAM10 mutant with a minimum activity in the absence of TEV that was significantly increased by TEV-mediated domain removal (Figure 4 and Figure 5).

Then, combining the expression of TEV-inducible ADAM10 activation mutants with a chemically activatable TEV, we controlled ADAM10 activity in living cells using the well-tolerated, clinically approved, blood–brain barrier-permeant drug rapamycin [53,54].

Moreover, we provided evidence that our bioengineering strategy to control ADAM10 was able to enhance non-amyloidogenic APP processing. If validated in future in vivo applications, our TEV-mediated modulation of ADAM10 activity could represent a novel therapeutic strategy for the treatment and prevention of AD. Indeed, in humans, mutations in ADAM10 reducing its activity were found to be associated with AD [55]. Conversely, the overexpression of wt ADAM10 in an AD mouse model alleviated cognitive deficits by reducing Aβ accumulation [15,56].

However, given that ADAM10 has multiple substrates, it is essential to emphasize that although ADAM10 overactivation would result in beneficial effects for AD, it can be detrimental to other diseases, such as cancer. For this reason, we developed a TEV-inducible ADAM10 mutant with minimal activity in the absence of TEV-mediated cleavage, and further studies are needed to render the action of ADAM10 specific for APP. An object of attention for ADAM10 substrate selection could be the role of the members of the tetraspanin (TSPAN) family [57]. The specificity of ADAM10 for the cleavage of some of its substrates without concomitantly affecting all the others could be obtained with concomitant overexpression of members of the TSPANC8 subgroup, with TSPAN15 potentially useful in promoting ADAM10-mediated APP cleavage in neurons [58].

Our bioengineering strategy presented here using a well-tolerated, mammalian-safe, and inducible TEV protease, once validated in further studies, could pave the way for developing novel ADAM10-based strategies requiring its precise and temporal activation. We have provided evidence that ADAM10 activity can be controlled without intervening with other molecules that facilitate structural activation, membrane expression, trafficking, and export, but by directly promoting ADAM10 cleavage in living cells.

AD drugs have almost exclusively sought to use molecules and antibodies targeted toward misfolded Aβ with the potential benefit to cover both familial and sporadic forms of AD [59,60]. AD drugs targeting misfolded Aβ failed to show benefit in large clinical trials involving patients with moderate AD [61]. However, very recently, an AD drug using humanized IgG1 monoclonal antibody to target misfolded Aβ resulted in delayed cognitive decline in persons with early AD [62]. Of note, in AD, the accumulation of Aβ following excessive amyloidogenic APP processing occurs with a parallel decrease in the neuroprotective and neurotrophic factor sAPPα. Thus, targeting Aβ accumulation without promoting the production of neuroprotective APP fragments could limit the clinical benefit. Once validated in vivo studies, our bioengineering strategy, leveraging the α-secretase-dependent cleavage of APP, precludes the amyloidogenic pathway of Aβ generation and, at the same time, increases the production of sAPPα; thus, it could be potentially used as a new strategy to delay or revert the pathological effects in AD.

## 4. Materials and Methods

### 4.1. DNA Plasmid Cloning

Mutation of the identified insertion TEVcs was introduced on the pRK5M-ADAM10 plasmid (addgene: 31717) through the Gibson Assembly reaction (Gibson Cloning MasterMix, NEB, Ipswich, MA, USA) following the manufacturer’s instructions. All the primers were designed with by SnapGene software (GSL Biotech, San Diego, CA, USA). The insertion of the T719A point mutation was introduced by PCR extension and ligation with T4 Polynucleotide Kinase (T4 PNK, NEB, Ipswich, MA, USA) and T4 DNA Ligase (NEB, Ipswich, MA, USA). Protein structure modeling was performed using AlphaFold2 as previously described [63]. Sequencing data were inspected using Sanger Sequencing and Fragment Analysis Software SeqScape of Applied Biosystems (ThermoFisher Scientific, Waltham, MA, USA) and assisted by SnapGene software (GSL Biotech, San Diego, CA, USA).

### 4.2. Cell Cultures and DNA Transfections

HEK293T cells were cultured at passage 4–20 in high-glucose Dulbecco’s modified Eagle medium (DMEM, Sigma-Aldrich, St. Louis, MO, USA) supplemented with 10% *v*/*v* fetal bovine serum (FBS, Sigma-Aldrich, St. Louis, MO, USA) without antibiotics and incubated at 37 °C temperature and 5% CO_2_ conditions as previously described [44]. For APP695 with Swedish mutation-overexpressing HEK293 cells, the cultured medium was supplemented with 150 µg/mL of G418 (ThermoFisher Scientific, Waltham, MA, USA). For shedding assay and Western blot experiments, 3 × 10^5^ cells were plated into 6-well plates. DNA plasmid vectors were transfected at 60–90% cell confluency with a DNA-PEI max mixture consisting of 2.5 µg total DNA along with 7.5 µg pH 7.3, polyethylenimine HCl max solution (PEI max, Polysciences, Warrington, PA, USA) in Opti-MEM Reduced-Serum Medium (ThermoFisher Scientific, Waltham, MA, USA).

### 4.3. Ectodomain Shedding the Assay 

For the ectodomain shedding assay after 24 h of transfection, cell medium was replaced with fresh Opti-MEM Reduced-Serum Medium for 4 h. In cells transfected with uniRapR-TEV, 7 µM rapamycin or 99% ethanol as vehicle was used. For colorimetric quantification of AP shedding, we followed a sensitive and semiquantitative method described in [64]. Briefly, cell medium was collected before that the cells were lysed with ice-cold lysis buffer (150 mM NaCl, 50 mM Tris-HCl  pH 7.4, 2 mM EDTA) containing 1% Triton X-100, 0.1% SDS, 10% glycerol, 1× cOmplete Ultra tablets protease inhibitor cocktail (Roche, Basel, Switzerland), 1  mM sodium orthovanadate (Sigma-Aldrich, St. Louis, MO, USA), 11 mM β-glycerolphosphate, 10  mM sodium fluoride (Sigma-Aldrich, St. Louis, MO, USA). Cell medium and cell lysate were mixed with 100 µL of a 2 mg/mL solution of the alkaline phosphatase substrate 4-nitrophenyl phosphate in 100 mM Tris-HCl, pH 9.5, 100 mM NaCl, 20 mM MgCl_2_. After incubation at 37 °C for 15–60 min, the A405 signal of AP activity was determined using a 96-well plate reader. Cells transfected with only BTC-AP plasmid reflect endogenous shedding levels. Values are subtracted from endogenous shedding levels and expressed as the ratio between AP activity in cell medium and AP total activity (cell medium + cell lysate). BTC-AP plasmid was a kind gift from Dr Carl Blobel and Dr Shigeki Higashiyama.

### 4.4. Western Blotting

Cell lysed supernatant was quantified for protein content (DC Protein Assay; Bio-Rad, Hercules, CA, USA). Cells were lysed with ice-cold lysis buffer (150 mM NaCl, 50 mM Tris-HCl  pH 7.4, 2 mM EDTA) containing 1% Triton X-100, 0.1% SDS, 10% glycerol, 1× cOmplete Ultra tablets protease inhibitor cocktail (Roche, Basel, Switzerland), 1  mM sodium orthovanadate (Sigma-Aldrich, St. Louis, MO, USA), 11 mM β-glycerolphosphate, 10  mM sodium fluoride (Sigma-Aldrich, St. Louis, MO, USA). Equal amounts of protein were diluted in Laemmli buffer, boiled, and resolved by SDS-PAGE. The primary antibodies were incubated for 1 h at room temperature (RT) or overnight at 4 °C and revealed with horseradish peroxidase-conjugated secondary antibodies (Cell Signaling, Danvers, MA, USA). Expression was evaluated and documented by using UVItec Cambridge Alliance.

### 4.5. Quantification of Soluble APP

HEK293 cells overexpressing APP695 Swedish mutation were transfected, as indicated before. After 24 h of transfection, the cell medium was replaced with fresh medium supplemented with 150 ug/mL of G418 (ThermoFisher Scientific, Waltham, MA, USA). After 24 h of transfection, the conditioned medium was collected and the cells lysed. Based on total protein content from lysed cells, an equal amount of conditioned medium was used for SDS-PAGE analyses.

### 4.6. Immunofluorescence Analyses

HEK293T cells transfected with Myc-tagged ADAM10 analogs were fixed with 10% formalin solution (pH 7.4 with NaOH) for 15 min at RT. After washing with PBS, cells were permeabilized with 0.3% Triton X-100 (Sigma-Aldrich, St. Louis, MO, USA) in PBS for 15 min and blocked with 0.3% BSA in PBS for 30 min at RT. Incubation with primary antibody was performed overnight at 4 °C in the blocking solution. The next day, the secondary antibody (Alexa Fluor488, ThermoFisher Scientific, Waltham, MA, USA) was incubated for 90 min at RT in PBS. Finally, cells were incubated with 4’,6-diamidino-2-phenylindole (DAPI, ThermoFisher Scientific, Waltham, MA, USA), 0.5 mg/mL in PBS for 10 min at RT and cells were then mounted on a microscope slide using the mounting medium ProLong Gold antifade reagent (Invitrogen, Waltham, MA, USA). Images were acquired by using a confocal laser scanning system (Nikon Ti-E, Confocal Head A1 MP, Tokyo, Japan) with a 60× oil-immersion objective lens. Each experiment was repeated at least three times with independent cell preparations.

### 4.7. Antibodies

The following antibodies were used: anti-Myc-Tag (9B11) antibody (Cell Signaling, Danvers, MA, USA), anti-HA-Tag (6E2) antibody (Cell Signaling, Danvers, MA, USA), anti-V5 antibody (Invitrogen, Waltham, MA, USA), anti-β-tubulin antibody (Cell Signaling, Danvers, MA, USA), APP Y188 (Abcam, Cambridge, UK), anti-6E10 antibody (BioLegend, San Diego, CA, USA).

### 4.8. Statistical Analysis

The statistical tests used (i.e., Student’s *t*-test, one-way ANOVA with Dunnett’s post hoc test comparisons) are indicated in the corresponding figure legends for each experiment. All statistical tests were two-tailed and the level of significance was set at 0.05. Results are shown as mean ± SEM.

## Data Availability

Additional datasets that support the finding of this study are available from the corresponding authors upon request.

## References

[1] Miller M.A., Meyer A.S., Beste M.T., Lasisi Z., Reddy S., Jeng K.W., Chen C.-H., Han J., Isaacson K., Griffith L.G. (2013). ADAM-10 and -17 regulate endometriotic cell migration via concerted ligand and receptor shedding feedback on kinase signaling. Proc. Natl. Acad. Sci. USA.

[2] Alabi R.O., Glomski K., Haxaire C., Weskamp G., Monette S., Blobel C.P. (2016). ADAM10-Dependent Signaling Through Notch1 and Notch4 Controls Development of Organ-Specific Vascular Beds. Circ. Res..

[3] Peschon J.J., Slack J.L., Reddy P., Stocking K.L., Sunnarborg S.W., Lee D.C., Russell W.E., Castner B.J., Johnson R.S., Fitzner J.N. (1998). An essential role for ectodomain shedding in mammalian development. Science.

[4] Lambrecht B.N., Vanderkerken M., Hammad H. (2018). The emerging role of ADAM metalloproteinases in immunity. Nat. Rev. Immunol..

[5] Pruessmeyer J., Ludwig A. (2009). The good, the bad and the ugly substrates for ADAM10 and ADAM17 in brain pathology, inflammation and cancer. Semin. Cell Dev. Biol..

[6] Weber S., Saftig P. (2012). Ectodomain shedding and ADAMs in development. Development.

[7] Hsia H.E., Tüshaus J., Brummer T., Zheng Y., Scilabra S.D., Lichtenthaler S.F. (2019). Functions of ‘A disintegrin and metalloproteases (ADAMs)’ in the mammalian nervous system. Cell Mol. Life Sci..

[8] Edwards D.R., Handsley M.M., Pennington C.J. (2008). The ADAM metalloproteinases. Mol. Aspects Med..

[9] Lammich S., Kojro E., Postina R., Gilbert S., Pfeiffer R., Jasionowski M., Haass C., Fahrenholz F. (1999). Constitutive and regulated alpha-secretase cleavage of Alzheimer’s amyloid precursor protein by a disintegrin metalloprotease. Proc. Natl. Acad. Sci. USA.

[10] Kuhn P.H., Wang H., Dislich B., Colombo A., Zeitschel U., Ellwart J.W., Kremmer E., Roßner S., Lichtenthaler S.F. (2010). ADAM10 is the physiologically relevant, constitutive alpha-secretase of the amyloid precursor protein in primary neurons. EMBO J..

[11] Lichtenthaler S.F. (2011). α-secretase in Alzheimer’s disease: Molecular identity, regulation and therapeutic potential. J. Neurochem..

[12] Kojro E., Fahrenholz F. (2005). The non-amyloidogenic pathway: Structure and function of alpha-secretases. Subcell Biochem..

[13] Manzine P.R., Pelucchi S., Horst M.A., Vale F.A., Pavarini S.C., Audano M., Mitro N., Di Luca M., Marcello E., Cominetti M.R. (2018). microRNA 221 Targets ADAM10 mRNA and is Downregulated in Alzheimer’s Disease. J. Alzheimers Dis..

[14] Yuan X.Z., Sun S., Tan C.C., Yu J.T., Tan L. (2017). The Role of ADAM10 in Alzheimer’s Disease. J. Alzheimers Dis..

[15] Postina R., Schroeder A., Dewachter I., Bohl J., Schmitt U., Kojro E., Prinzen C., Endres K., Hiemke C., Blessing M. (2004). A disintegrin-metalloproteinase prevents amyloid plaque formation and hippocampal defects in an Alzheimer disease mouse model. J. Clin. Invest..

[16] Lichtenthaler S.F., Haass C. (2004). Amyloid at the cutting edge: Activation of alpha-secretase prevents amyloidogenesis in an Alzheimer disease mouse model. J. Clin. Invest..

[17] Saraceno C., Marcello E., Di Marino D., Borroni B., Claeysen S., Perroy J., Padovani A., Tramontano A., Gardoni F., Di Luca M. (2014). SAP97-mediated ADAM10 trafficking from Golgi outposts depends on PKC phosphorylation. Cell Death Dis..

[18] Marcello E., Saraceno C., Musardo S., Vara H., De La Fuente A.G., Pelucchi S., Di Marino D., Borroni B., Tramontano A., Perez-Otano I. (2013). Endocytosis of synaptic ADAM10 in neuronal plasticity and Alzheimer’s disease. J. Clin. Invest..

[19] Borcel E., Palczynska M., Krzisch M., Dimitrov M., Ulrich G., Toni N., Fraering P.C. (2016). Shedding of neurexin 3β ectodomain by ADAM10 releases a soluble fragment that affects the development of newborn neurons. Sci. Rep..

[20] Trotter J.H., Hao J., Maxeiner S., Tsetsenis T., Liu Z., Zhuang X., Südhof T.C. (2019). Synaptic neurexin-1 assembles into dynamically regulated active zone nanoclusters. J. Cell Biol..

[21] Seegar T.C.M., Killingsworth L.B., Saha N., Meyer P.A., Patra D., Zimmerman B., Janes P.W., Rubinstein E., Nikolov D.B., Skiniotis G. (2017). Structural Basis for Regulated Proteolysis by the α-Secretase ADAM10. Cell.

[22] McCulloch D.R., Akl P., Samaratunga H., Herington A.C., Odorico D.M. (2004). Expression of the disintegrin metalloprotease, ADAM-10, in prostate cancer and its regulation by dihydrotestosterone, insulin-like growth factor I, and epidermal growth factor in the prostate cancer cell model LNCaP. Clin. Cancer Res..

[23] Wang C.-Y., Zheng W., Wang T., Xie J.-W., Wang S.-L., Zhao B.-L., Teng W.-P., Wang Z.-Y. (2011). Huperzine A activates Wnt/β-catenin signaling and enhances the nonamyloidogenic pathway in an Alzheimer transgenic mouse model. Neuropsychopharmacology.

[24] Lee H.R., Shin H.K., Park S.Y., Kim H.Y., Lee W.S., Rhim B.Y., Hong K.W., Kim C.D. (2014). Cilostazol suppresses β-amyloid production by activating a disintegrin and metalloproteinase 10 via the upregulation of SIRT1-coupled retinoic acid receptor-β. J. Neurosci. Res..

[25] Prinzen C., Müller U., Endres K., Fahrenholz F., Postina R. (2005). Genomic structure and functional characterization of the human ADAM10 promoter. FASEB J..

[26] Lammich S., Kamp F., Wagner J., Nuscher B., Zilow S., Ludwig A.K., Willem M., Haass C. (2011). Translational repression of the disintegrin and metalloprotease ADAM10 by a stable G-quadruplex secondary structure in its 5’-untranslated region. J. Biol. Chem..

[27] Saint-Pol J., Eschenbrenner E., Dornier E., Boucheix C., Charrin S., Rubinstein E. (2017). Regulation of the trafficking and the function of the metalloprotease ADAM10 by tetraspanins. Biochem. Soc. Trans..

[28] Bleibaum F., Sommer A., Veit M., Rabe B., Andrä J., Kunzelmann K., Nehls C., Correa W., Gutsmann T., Grötzinger J. (2019). ADAM10 sheddase activation is controlled by cell membrane asymmetry. J. Mol. Cell Biol..

[29] Kojro E., Postina R., Buro C., Meiringer C., Gehrig-Burger K., Fahrenholz F. (2006). The neuropeptide PACAP promotes the alpha-secretase pathway for processing the Alzheimer amyloid precursor protein. FASEB J..

[30] Van Wart H.E., Birkedal-Hansen H. (1990). The cysteine switch: A principle of regulation of metalloproteinase activity with potential applicability to the entire matrix metalloproteinase gene family. Proc. Natl. Acad. Sci. USA.

[31] Lum L., Reid M.S., Blobel C.P. (1998). Intracellular maturation of the mouse metalloprotease disintegrin MDC15. J. Biol. Chem..

[32] Anders A., Gilbert S., Garten W., Postina R., Fahrenholz F. (2001). Regulation of the alpha-secretase ADAM10 by its prodomain and proprotein convertases. FASEB J..

[33] Moss M.L., Bomar M., Liu Q., Sage H., Dempsey P., Lenhart P.M., Gillispie P.A., Stoeck A., Wildeboer D., Bartsch J.W. (2007). The ADAM10 prodomain is a specific inhibitor of ADAM10 proteolytic activity and inhibits cellular shedding events. J. Biol. Chem..

[34] Wong E., Maretzky T., Peleg Y., Blobel C.P., Sagi I. (2015). The Functional Maturation of A Disintegrin and Metalloproteinase (ADAM) 9, 10, and 17 Requires Processing at a Newly Identified Proprotein Convertase (PC) Cleavage Site. J. Biol. Chem..

[35] Deng W., Cho S., Su P.C., Berger B.W., Li R. (2014). Membrane-enabled dimerization of the intrinsically disordered cytoplasmic domain of ADAM10. Proc. Natl. Acad. Sci. USA.

[36] Xu P., Liu J., Sakaki-Yumoto M., Derynck R. (2012). TACE activation by MAPK-mediated regulation of cell surface dimerization and TIMP3 association. Sci. Signal..

[37] Cissé M., Duplan E., Guillot-Sestier M.V., Rumigny J., Bauer C., Pages G., Orzechowski H.D., Slack B.E., Checler F., Vincent B. (2011). The extracellular regulated kinase-1 (ERK1) controls regulated alpha-secretase-mediated processing, promoter transactivation, and mRNA levels of the cellular prion protein. J. Biol. Chem..

[38] Díaz-Rodríguez E., Montero J.C., Esparís-Ogando A., Yuste L., Pandiella A. (2002). Extracellular signal-regulated kinase phosphorylates tumor necrosis factor alpha-converting enzyme at threonine 735: A potential role in regulated shedding. Mol. Biol. Cell..

[39] Soond S.M., Everson B., Riches D.W.H., Murphy G. (2005). ERK-mediated phosphorylation of Thr735 in TNFalpha-converting enzyme and its potential role in TACE protein trafficking. J. Cell Sci..

[40] Maretzky T., Evers A., Le Gall S., Alabi R.O., Speck N., Reiss K., Blobel C.P. (2015). The cytoplasmic domain of a disintegrin and metalloproteinase 10 (ADAM10) regulates its constitutive activity but is dispensable for stimulated ADAM10-dependent shedding. J. Biol. Chem..

[41] Marcello E., Gardoni F., Di Luca M., Pérez-Otaño I. (2010). An arginine stretch limits ADAM10 exit from the endoplasmic reticulum. J. Biol. Chem..

[42] Tippmann F., Hundt J., Schneider A., Endres K., Fahrenholz F. (2009). Up-regulation of the alpha-secretase ADAM10 by retinoic acid receptors and acitretin. FASEB J..

[43] Zheng Y., Schlondorff J., Blobel C.P. (2002). Evidence for regulation of the tumor necrosis factor alpha-convertase (TACE) by protein-tyrosine phosphatase PTPH1. J. Biol. Chem..

[44] Renna P., Ripoli C., Dagliyan O., Pastore F., Rinaudo M., Re A., Paciello F., Grassi C. (2022). Engineering a switchable single-chain TEV protease to control protein maturation in living neurons. Bioeng Transl Med..

[45] Menon P.K., Koistinen N.A., Iverfeldt K., Ström A.L. (2019). Phosphorylation of the amyloid precursor protein (APP) at Ser-675 promotes APP processing involving meprin β. J Biol Chem..

[46] Vella L.J., Cappai R. (2012). Identification of a novel amyloid precursor protein processing pathway that generates secreted N-terminal fragments. FASEB J..

[47] Wetzel S., Seipold L., Saftig P. (2017). The metalloproteinase ADAM10: A useful therapeutic target?. Biochim. Biophys. Acta Mol. Cell Res..

[48] Smith T.M., Tharakan A., Martin R.K. (2020). Targeting ADAM10 in Cancer and Autoimmunity. Front Immunol..

[49] Sakamoto K., Jin S.-P., Goel S., Jo J.-H., Voisin B., Kim D., Nadella V., Liang H., Kobayashi T., Huang X. (2021). Disruption of the endopeptidase ADAM10-Notch signaling axis leads to skin dysbiosis and innate lymphoid cell-mediated hair follicle destruction. Immunity.

[50] Endres K., Deller T. (2017). Regulation of Alpha-Secretase ADAM10 In vitro and In vivo: Genetic, Epigenetic, and Protein-Based Mechanisms. Front Mol. Neurosci..

[51] Tosetti F., Alessio M., Poggi A., Zocchi M.R. (2021). ADAM10 Site-Dependent Biology: Keeping Control of a Pervasive Protease. Int. J. Mol. Sci..

[52] Reiss K., Leitzke S., Seidel J., Sperrhacke M., Bhakdi S. (2022). Scramblases as Regulators of Proteolytic ADAM Function. Membranes.

[53] Tramutola A., Lanzillotta C., Barone E., Arena A., Zuliani I., Mosca L., Blarzino C., Butterfield D.A., Perluigi M., Di Domenico F. (2018). Intranasal rapamycin ameliorates Alzheimer-like cognitive decline in a mouse model of Down syndrome. Transl. Neurodegener..

[54] Selvarani R., Mohammed S., Richardson A. (2021). Effect of rapamycin on aging and age-related diseases-past and future. Geroscience.

[55] Colciaghi F., Borroni B., Pastorino L., Marcello E., Zimmermann M., Cattabeni F., Padovani A., Di Luca M. (2002). [alpha]-Secretase ADAM10 as well as [alpha]APPs is reduced in platelets and CSF of Alzheimer disease patients. Mol. Med..

[56] Schmitt U., Hiemke C., Fahrenholz F., Schroeder A. (2006). Over-expression of two different forms of the alpha-secretase ADAM10 affects learning and memory in mice. Behav. Brain Res..

[57] Harrison N., Koo C.Z., Tomlinson M.G. (2021). Regulation of ADAM10 by the TspanC8 Family of Tetraspanins and Their Therapeutic Potential. Int. J. Mol. Sci..

[58] Prox J., Willenbrock M., Weber S., Lehmann T., Schmidt-Arras D., Schwanbeck R., Saftig P., Schwake M. (2012). Tetraspanin15 regulates cellular trafficking and activity of the ectodomain sheddase ADAM10. Cell Mol. Life Sci..

[59] Tolar M., Abushakra S., Hey J.A., Porsteinsson A., Sabbagh M. (2020). Aducanumab, gantenerumab, BAN2401, and ALZ-801-the first wave of amyloid-targeting drugs for Alzheimer’s disease with potential for near term approval. Alzheimers Res Ther..

[60] Chegaev K., Federico A., Marini E., Rolando B., Fruttero R., Morbin M., Rossi G., Fugnanesi V., Bastone A., Salmona M. (2015). NO-donor thiacarbocyanines as multifunctional agents for Alzheimer’s disease. Bioorg Med. Chem..

[61] Panza F., Lozupone M., Logroscino G., Imbimbo B.P. (2019). A critical appraisal of amyloid-β-targeting therapies for Alzheimer disease. Nat. Rev. Neurol..

[62] Van Dyck C.H., Swanson C.J., Aisen P., Bateman R.J., Chen C., Gee M., Kanekiyo M., Li D., Reyderman L., Cohen S. (2022). Lecanemab in Early Alzheimer’s Disease. N Engl. J. Med..

[63] Mirdita M., Schütze K., Moriwaki Y., Heo L., Ovchinnikov S., Steinegger M. (2022). ColabFold: Making protein folding accessible to all. Nat. Methods.

[64] Sahin U., Weskamp G., Zheng Y., Chesneau V., Horiuchi K., Blobel C.P. (2006). A sensitive method to monitor ectodomain shedding of ligands of the epidermal growth factor receptor. Methods Mol. Biol..

